# Developmental Exposures to Three Mammalian Teratogens Produce Dysmorphic Phenotypes in Adult *Caenorhabditis elegans*

**DOI:** 10.3390/toxics13070589

**Published:** 2025-07-14

**Authors:** Piper Reid Hunt, Martine Ferguson, Nicholas Olejnik, Jeffrey Yourick, Robert L. Sprando

**Affiliations:** 1 U.S. Food and Drug Administration, Human Foods Program, Office of Chemistry and Toxicology, Laurel, MD 20708, USA; 2 U.S. Food and Drug Administration, Human Foods Program, Office of Surveillance Strategy & Risk Prioritization, Division of Surveillance & Data Integration, College Park, MD 20740, USA

**Keywords:** teratogen, morphometry, developmental toxicity, chemical screening, alternative toxicity test method, small model organism

## Abstract

Efficient new methods are needed to support initiatives to reduce, refine, and/or replace toxicity testing in vertebrates. 5-fluorouracil (5FU), hydroxyurea (HU), and ribavirin (RV) are mammalian teratogens. Skeletal, endocrine organ, and cardiac effects are often associated with teratogenesis, and a simple nematode like *C. elegans* lacks these systems. However, many genetic pathways required for mammalian morphogenesis have at least some conserved elements in this small, invertebrate model. The *C. elegans* lifecycle is 3 days. The effects of 5FU, HU, and RV on the *C. elegans* morphology were evaluated on day 4 post-initiation of the feeding after hatching for continuous and 24 h (early-only) developmental exposures. Continuous exposures to 5FU and HU induced increases in the incidences of abnormal gonadal structures that were significantly reduced in early-only exposure groups. The incidence of prolapse increased with continuous 5FU and HU exposures and was further increased in early-only exposure groups. Intestinal prolapse through the vulval muscle in *C. elegans* may be related to reported 5FU and HU effects on skeletal muscle and the gastrointestinal tract in mammals. Continuous RV exposures induced a phenotype lacking a uterus and gonad arms, as well as vulval anomalies that were largely, but not completely, reversed with early-only exposures, which is consistent with reported reversible reproductive tract anomalies after an RV exposure in mammals. These findings suggest that *C. elegans* can be used to detect the hazard risk from chemicals that adversely affect conserved pathways involved in organismal morphogenesis, but to determine the fit-for-purpose use of this model in chemical safety evaluations, further studies using larger and more diverse chemical test panels are needed.

## 1. Introduction

Teratogens induce congenital malformations and disorders when exposures occur at specific times during development. In different mammalian species, there are significant differences in the sensitivity to many teratogens as well as differences in induced gross dysmorphic features, and no single non-primate mammalian species has been shown to more reliably predict human teratogenic effects than any other [1,2]. This is likely due to species differences in pharmacokinetics, the species-specific timing of critical periods of developmental susceptibility, and strategies for maternal–embryonic exchange; these differences indicate that, relative to the malformation data alone, integrating reproductive and developmental effects data from a variety of models and endpoints will better reflect the chemical risk to humans [1,2,3,4,5].

Legislation in the U.S. and E.U. has been implemented to limit or replace toxicity testing in vertebrate animals [6,7]. Organismal development encompasses many complex processes that cannot be fully modeled in vitro; however, the broad genetic conservation of developmental regulation indicates that test data from invertebrate organisms can be used to help fill chemical hazard data gaps [8]. *C. elegans* are microscopic, non-pathogenic nematodes that can be maintained and tested using standard in vitro laboratory equipment and at a low-cost relative to in vivo testing in vertebrates [9]. While the timing of *C. elegans’* embryonic first cleavage, gastrulation, and organogenesis differ greatly from mammals, many of the genetic, cellular, and tissue events involved in these processes are conserved [8,10]. Additionally, some *C. elegans* tissue specification and neuronal migration events occur after hatching [11], allowing for chemical effects on some morphological processes to be evaluated separately from maternal exposures.

Several mammalian teratogens have been shown to arrest the growth of *C. elegans* embryos, delay juvenile development, and/or reduce the brood size [12,13,14]. Early exposures to cadmium, ethanol, ketamine, mercury, and methamphetamine are associated with developmental malformations in mammals and *C. elegans* [15,16,17,18]; however, identified published *C. elegans* studies that noted chemically induced malformations did not provide quantification or incidence data on specific phenotypes. Previously, we found that mammalian teratogens 5-fluorouracil, hydroxyurea, and ribavirin cause developmental delays and altered locomotor activity levels [19]. In that study, morphologic and reproductive anomalies were observed and noted but not quantified. Here, the adult morphology and the reversibility of effects from developmental exposures to these three chemicals were quantified and evaluated.

## 2. Materials and Methods

### 2.1. C. elegans Culture Maintenance and Chemical Exposures

**Figure 1:**
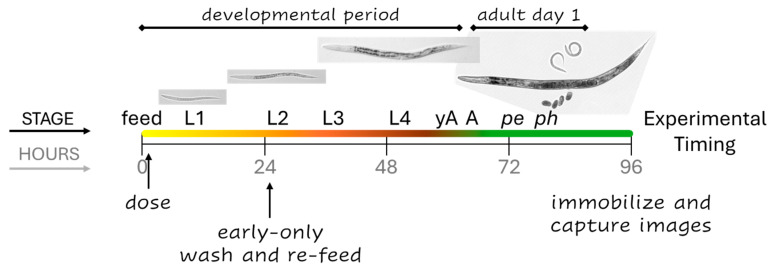
The experimental outline. In control cohorts, the four larval stages (L1–4) progress over approximately 58 h from the first feeding at time zero (feed). Young adults (yAs) have a uterus and oocytes, but once an oocyte passes through the spermatheca into the uterus and is fertilized, *C. elegans* are considered adults (A). The majority of control parental cohorts begin to lay progeny eggs (*pe*) around the 70 h mark, and progeny begin to hatch (*ph*) 5 to 8 h after that. Continuous exposures began at dosing and continued until imaging four days later. Early-only exposures were for the first 24 h of development.

N2 wild-type *C. elegans* were purchased from the *Caenorhabditis* Genetics Center, which is funded by NIH Office of Research Infrastructure Programs (P40 OD010440). Healthy, well-fed cultures suitable for toxicity testing were maintained and exposed in *C. elegans* Habitation Medium (CeHM) as previously described [20]. CeHM allows for *C. elegans* growth rates comparable to those found using *E. coli* as a feeder organism, but without the complication of a secondary metabolism and toxicity profile within the test system [21]. Nutrient media ingredients and test articles were purchased from Sigma-Aldrich (Saint Louis, MO, USA). Four independent experiments were conducted for each of the three water soluble test articles using autoclaved MilliQ-filtered tap water as the solvent and control. Age-synchronized cohorts were generated only from healthy, well-fed cultures without dauers. Each independent experiment consisted of a unique *C. elegans* cohort and a freshly prepared, unique set of dosing solutions. As determined previously, the three highest sublethal concentrations of test articles that still allowed for at least partially synchronous developmental timing within exposed populations were utilized [19]. For continuous exposures, cohorts were isolated, dosed, and maintained in 12-well plates at 20 °C as previously described [22]. For early-only exposures, *C. elegans* cohorts were quickly washed twice and then re-fed CeHM at 24.5 h ± 30 min for a maximum of 10min without food. The timing of *C. elegans* development, exposures, and experimental steps is summarized in Figure 1.

### 2.2. Imaging and Gross Phenotype Analysis

On day four post-L1 feeding and dosing, when control populations were adults with early-stage progeny, *C. elegans* populations were immobilized with sodium azide directly in CeHM. Washing and pipetting onto slides was not performed due to the fragility of some phenotypes, especially prolapse and ‘sick’ individuals (described in Section 3), which had a tendency to fall apart with handling. Plates were transferred to a Keyence (Itasca, IL, USA) automated stage microscope for image capture and later morphology assessment. One replicate well was imaged per condition per experiment and the *C. elegans* in the top half of each imaged well were later evaluated for morphology. A 300-individual minimum (excluding progeny) for analysis per condition per experiment was set, and due to random placement, for some wells a few contiguous images covering slightly over half the well were included in the analysis to reach the 300 minimum. Note that light conditions vary across a well, and *C. elegans* closest to the edge that could not be adequately assessed for morphology were not included in the assessment. L1- and L2-sized worms were considered progeny. Example images in figures were edited in Adobe Photoshop to remove distracting background debris. A folder with the original images used in figures along with the complete image sets generated for this study were deposited at dataDryad.org, Dataset DOI: 10.5061/dryad.j3tx95xs9.

### 2.3. Statistical Analysis

Four independent experiments were conducted for each chemical. For analyses, the data is presented as the percentage of *C. elegans* counted in a single well with a given phenotype, with a minimum of 300 worms analyzed per condition per experiment. The percentages of worms with a defined condition were compared both across doses within group (continuous or early-only) and across group within dose using a Bayesian generalized linear model assuming a skew normal distribution for the response. Posterior predicted means and 95% credible intervals on the differences in these means were then computed, with intervals not including zero indicating significant differences. The Bayesian model was run using the brm in the brms R package version 4.4.1 [23], and posterior predicted means were compared using the emmeans and pairs functions in the emmeans R package 4.4.1 [24].

## 3. Results

### 3.1. Morphometry Methods and Control Findings

**Figure 2:**
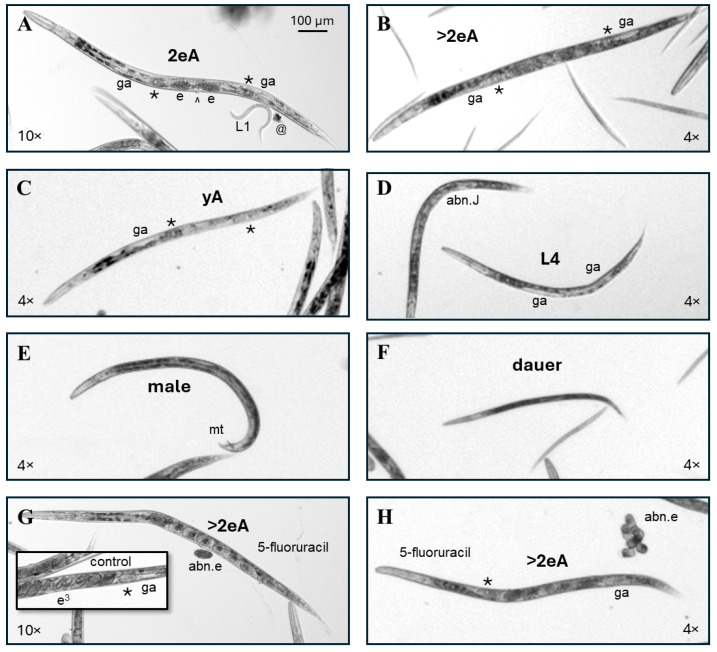
Examples of *C. elegans* phenotypes scored as normal. (**A**) A micrograph captured with a 10× objective of a control adult *C. elegans* with two fertilized eggs (e) in its uterus (2eA) and visible primary oocytes (*) and gonad arms (ga) on either side of the central vulva (^). A first-larval-stage progeny (L1) and a bit of debris (@) are also labeled. (**B**) A micrograph captured with a 4× objective of a control adult *C. elegans* with more than two fertilized eggs (e) in its uterus (>2eA). The 4× image is less crisp but still allows for a sufficient visualization of key features for scoring. (**C**) The control young adult (yA) with visible primary oocytes (*) and gonad arms (ga) on either side of the central uterus but no internal fertilized eggs. (**D**) In the center of the image is a 5-fluorouracil-exposed individual scored as a normal L4 due to the body size and apparently normal L4 gonad arm development. The abnormal juvenile (abn.J) on the left has disorganized gonadal structures. (**E**) An adult male *C. elegans* with its male tail (mt) labeled. (**F**) Dauers are darker and thinner than their growing counterparts of similar lengths. (**G**,**H**). The pictured *C. elegans* with a normal adult morphology (>2eA), but abnormal internal and laid eggs (abn.e) were continuously exposed to 5-fluorouracil from the first feeding after hatching. The inset in (**G**) shows trifold embryos (e3) developing in the uterus of a control adult for comparison. All images were enlarged to correspond to the upper left 100 µm scale bar.

For this study, the entire well for each condition was recorded in overlapping micrographs imaged with a 4× objective, while the center of the well was captured in 10× objective micrographs. During the analysis, it became clear that damaged phenotypes and progeny tended to aggregate towards the edges of the wells; therefore, the 4× images were utilized for the morphology evaluation, and the 10× images were used as examples. Figure 2 provides examples of phenotypes scored as normal. The example control adult imaged with a 10× objective to show the detail has two internal fertilized embryos (e) on either side of the vulva (^), while primary oocytes (*) and gonad arms (ga) are visible on either side of the central uterus (Figure 2A). The example control adult captured with a 4× objective (Figure 2B) is less clear than the 10× image, but several developing eggs in the uterus at the center of the body, primary oocytes, and gonad arms, along with a grossly normal head, intestinal, and tail morphology, are still distinguishable. To evaluate the maturity, adults were divided into groups with either one or two internal fertilized eggs (1–2eA) or more than two eggs (>2eA). In retrospect, this may not have been an ideal cut-off as many adults with only two internal eggs were larger than some adults with three or four internal eggs but had empty uterine regions, indicating that they had recently laid eggs. In future studies where a measure of adult maturity is required, a cut-off of four or more internal eggs may prove more informative.

Individuals were scored as a normal young adult (yA, Figure 2C) if they a. had no internal fertilized embryos, b. were slightly smaller than adults with internal eggs, and c. had at least two clearly visible yA features, such as two gonad arms taking up approximately three quarters of the length of the intestine, a visible primary oocyte, and/or a yA vulval structure. Individuals scored as normal fourth-larval-stage juveniles (L4, center Figure 2D) were slightly smaller than yAs, with clearly visible gonad arms taking up approximately two thirds the length of the intestine and/or visible L4 vulval features (reviewed in [25]). The other individual in Figure 2D was scored as an abnormal juvenile (abn.J) because it was about the size of a yA, but without a visible uterus or oocytes, and because the gonadal region appears disordered.

*C. elegans* males are XO, with five autosomes and a single X chromosome [26], and have a distinctive male tail (mt, Figure 2E). Males are created by nondisjunction—the failure of paired homologous chromosomes to accurately separate during cell division [27]. Nondisjunction can occur spontaneously or be chemically induced [28]. Across 12 independent experiments with 2 sets of controls each (one for continuous and one for early-only exposures), a total of 9150 control individuals were scored in this study, and 8 of those were males. Therefore, the frequency of males for cultures grown in CeHM was about one male per thousand, which is consistent with previous reports from *C. elegans* maintained on agar with *E. coli* as a feeder organism [27,29]. A total of 15 males were counted among 28,178 exposed individuals scored, indicating that 5FU, HU, and RV did not increase the incidence of males, which is consistent with toxicity mechanisms for these three chemicals being directed at nucleic acid synthesis and repair rather than microtubule function or other cellular machinery critical to chromosome segregation.

Dauer larvae are rarely seen in well-fed and maintained CeHM cultures. Dauers release pheromones that increase the stress resistance gene expression of other *C. elegans* in the same culture [30,31] and can thereby alter population responses to toxicants. Therefore, for toxicology purposes, cultures that contain dauers prior to chemical exposure should be discarded. Within this CeHM liquid nutrient media culture maintenance and exposure system, increases in the percentage of dauers in chemical-exposed populations are an indication of adverse effects, while dauer increases in control populations indicate poor culture handling and the exclusion of that experiment from findings. *C. elegans* dauers are approximately the length of third-larval-stage (L3) juveniles but are thinner and darker with less pale gonadal material than normal L3s (Figure 2F).

The 5FU-exposed individuals pictured in Figure 2G,H appear to have a fully normal adult morphology and were categorized as normal >2eA but produced abnormal internal and laid eggs (abn.e). For comparison, the inset shows a *C. elegans* control uterus with trifold embryos (e^3^) at the center and less mature embryos on either side (inset, Figure 2G). If a single feature was out of focus, such as the tail (Figure 2H) or part of the head, that feature was presumed to be normal; however, if a major portion of the body was out of focus, the worm was included in scoring only if it was clearly evident that it fit into a specific abnormal phenotype category.

**Figure 3:**
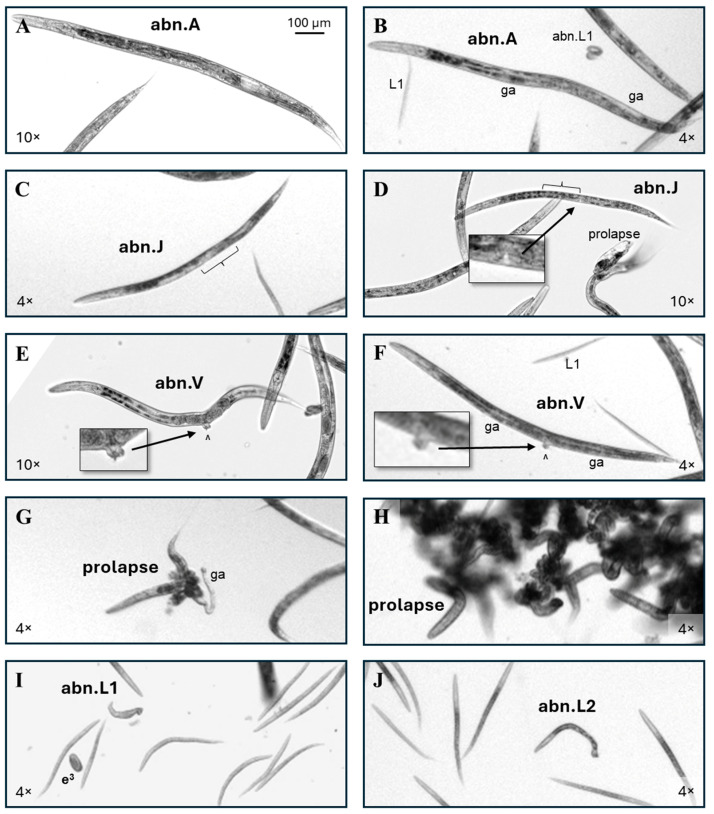
Abnormal phenotype examples. (**A**) Adult-sized individuals with abnormal reproductive structures but otherwise normal features were scored as an abnormal gonad adult (abn.A). (**B**) This adult has a grossly normal head, intestine, and tail morphology, but while there is evidence of at least one gonad arm (ga), there is no evidence of oocytes or internal fertilized eggs, so it was scored as an abn.A. Scoring examples of a normal and abnormal L1 are also labeled. (**C**) The central gonadal region of this late L4-sized 5FU-exposed worm appears disordered (bracket), putting it in the abnormal juvenile (abn.J) category. (**D**) A 5FU-exposed *C. elegans* with an L4 vulval structure (inset), but an apparently absent anterior gonad arm (bracket), is also an abn.J. (**E**,**F**) Examples of individuals with vulval (^) abnormalities (abn.V). (**G**) An example of a prolapsed individual where an intact gonad arm has also been pushed out of the body through the vulva. (**H**) The tangled intestines of prolapsed individuals frequently lead to their presentation as a clump of worms. (**I**) An example of an individual scored as an abnormal L1 (abn.L1) due to its length, with a laid trifold embryo egg (e^3^) also labeled. (**J**) An abnormal L2 (abn.L2). Non-inset 4× and 10× images were enlarged to correspond to the upper left 100 µm scale bar.

Common abnormal phenotypes in this study included those scored as an abnormal gonad adult (abn.A) with internal reproductive tract abnormalities in the absence of visible head, intestinal, vulval, or tail abnormalities (Figure 3A,B). Most abnormal juveniles (abn.Js) scored in this study were approximately L4-sized with disordered gonadal regions (bracket, Figure 3C) or more rarely an apparently missing single gonad arm (bracket, Figure 3D). A few adult-sized individuals with L4 vulval structures and without a visible uterus or oocytes were also scored as abn.Js. Individuals with a significantly protruding or, more rarely, jagged shaped vulva were scored as an abn.V, regardless of the presence (Figure 3E) or absence of internal eggs (Figure 3F). The pale regions on either side of the vulva (^) in Figure 3F indicate that this individual has gonad arms (ga), though they are short and indistinct for an adult. Among controls, the incidence of a visible abnormally shaped or protruding vulva was just under one per thousand (Table 1).

Prolapsed individuals had the intestine and/or uterus pushed out of the body through the vulva. In rare cases, a gonad arm (ga) was also visible outside the body (Figure 3G). Frequently, the tangled intestines of multiple prolapsed individuals caused them to form a clump (Figure 3H). The incidence of prolapse among control adults in this dataset was 1 in 400. Out of over 37,000 individual worms scored in this study, only 1 had an intestinal prolapse through the anus.

Worms the size of L1s and L2s were counted as progeny. Abnormal L1s (abn.L1, Figure 3I) and abnormal L2s (abn.L2, Figure 3J) were grouped together, irrespective of abnormality. In this study there was no difference between the control and any exposure group in the ratio of the abnormal progeny to the parental cohort. There is a possibility that the abn.L1 pictured (Figure 3I) is actually a member of the parental cohort due to its unusual width, but for this to be determined imaging would need to have been conducted prior to progeny hatching, and the phenotype was too rare for the chemicals in this study for the additional step to be informative.

**Figure 4:**
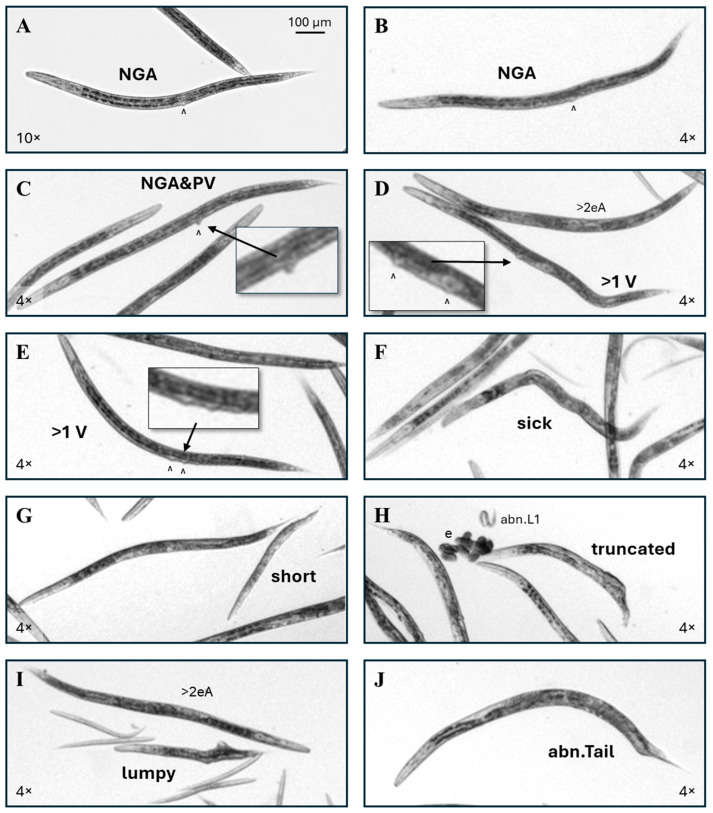
More abnormal phenotype examples. (**A**,**B**) Examples of ribavirin-exposed *C. elegans* without a visible uterus or gonad arms (NGA). (**C**) This individual lacks gonad arms and also has a protruding vulva (NGA&PV). (**D**) This was the single multi-vulval (>1 V) control *C. elegans* identified in this study, with the additional vulva (^) anterior to the normal vulval position. (**E**) A typical example of a ribavirin-exposed worm with >1 vulva. (**F**) Worms that had a pale or disordered internal appearance and >3 body bends, with or without visible internal gonadal or intestinal dysmorphy, but no other categorized gross anomaly, were scored as ‘sick’. (**G**) Unusually short worms with near-normal adult widths were scored as ‘short’. (**H**) An example of a truncated phenotype. (**I**) The ‘lumpy’ phenotype had outgrowths from the body. (**J**) An example of an abnormal tail (abn.Tail) is shown. Non-inset 4× and 10× images were enlarged to correspond to the upper left 100 µm scale bar.

Adult- or yA-sized individuals without a uterus or visible gonad arms, with or without a line of pale material extending anteriorly or posteriorly from the vulva, were scored as ‘no gonad arms’ (NGA, Figure 4A,B). Two NGA individuals were identified among all control cohorts, for an incidence of about one in five thousand. Adult-or yA-sized individuals with a significantly protruding vulva that also lacked visible gonad arms were scored as NGA&PV (Figure 4C). No NGA&PV individuals were identified among 9150 scored control *C. elegans*.

For this study, the multi-vulval or *muv* phenotype was categorized as >1 V. Of the 9150 scored control individuals, only one had a >1 V phenotype. Normally, the vulva is located at the approximate midpoint of the length of the intestine. In the single identified control >1 V individual, the additional vulva was anterior to and distant from the normal vulval position (Figure 4D). In ribavirin-exposed >1 V individuals, the additional vulva was usually positioned either posterior to and near the normal vulval location, or the two vulva were on either side of the normal vulval position (Figure 4E). Individuals with a disordered or pale intestine and abnormal posture, usually with four or more body bends, were scored as ‘sick’ (Figure 4F). The incidence of sick individuals in control cohorts was 1 in 300.

Worms scored as short had a range of lengths, but all were closer in width to normal adults than to developing *C. elegans* of corresponding lengths (Figure 4G). Truncated worms appeared to be cut somewhere in the middle rather than have produced an abnormal tail (Figure 4H). Note that this phenotype could be an artifact of the bleaching procedure in the egg isolation process for cohort generation. Regardless of the length, worms with outgrowths from the body were scored as ‘lumpy’ (Figure 4I). Head and tail (abn.Tail, Figure 4J) abnormalities were rare in this study, with 3 of 37,328 total scored worms having abnormal heads and 12 with abnormal tails. The incidences of these rare phenotypes are summarized in Table 1. No *C. elegans* with internally hatched eggs (the ‘bag of worms’ or *bow* phenotype), or intestinal stenosis as seen with the cadmium exposure [32], were identified in this study.

**Figure 5:**
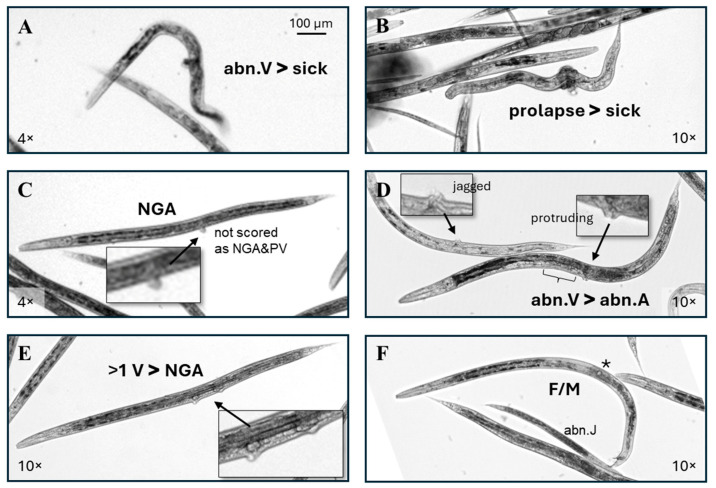
Scoring examples. (**A**,**B**) These individuals have > 3 body bends and the disordered internal appearance used for a categorization as sick, but the large protruding vulva put (**A**) in the abn.V category, and the material circling the midpoint put in (**B**) in the prolapse category. (**C**) If, on examination at a high magnification, a potential protruding vulva did not have a similar pixel intensity with adjacent tissue as in this example, it was assumed to be debris, regardless of the placement in the vulval region. (**D**) Two individuals that would have been scored as an abn.A without the visible vulval abnormalities. The (bracket) indicates a missing anterior half of the uterus. (**E**) This RV-exposed individual lacks gonad arms, but the presence of more than one vulva puts it in the >1 V category. (**F**) A rare RV-exposed *C. elegans* with both an oocyte (*) and a male tail (F/M).

The phenotype scoring priority was multi-vulval (>1 V) > lumpy > prolapse > short = truncated > no gonad arms (NGA) = abn.V > sick > abn.A. Figure 5A shows an example of an individual that would have been scored as sick, but an extreme protruding vulva put it in the abn.V category. Individuals scored as prolapse frequently also had a sick (Figure 5B) or short (Figure 3G) phenotype. To reduce the chances of debris contributing to the scoring, for each individual scored as having a vulval abnormality, the image was enlarged and examined to verify that the pixel intensity of the presumed vulval tissue was consistent with adjacent tissues. If a conclusion of contiguous tissue was questionable, the material was presumed to be debris, regardless of its shape or positioning (Figure 5C). The example of a yA-sized worm with short gonad arms and no visible oocytes or uterus shown in Figure 5D (left) would have been scored as an abn.A, but the jagged vulva put it in the abn.V category. In contrast to the large protruding vulva pictured in Figure 3E,F and Figure 5A, the protruding vulva example in Figure 5D (right) is on the smaller side of abnormal. The pictured individual also appears to lack the anterior half of the uterus (bracket) and so would have been scored as an abn.A without the protruding vulva (Figure 5D).

Individuals scored as >1 V frequently, but not always, also lacked gonad arms (Figure 4E and Figure 5E). Two individuals with both a visible oocyte (*) and a mail tail (mt) (F/M, Figure 5F) were identified in this dataset, both in a single early-only RV exposure cohort. The >1 V and F/M phenotypes were more difficult to identify at a low magnification than the other phenotypes scored in this study. The evaluation of images captured with a 10× objective rather than 4× may have increased the detection of these two phenotypes but would also have increased the required imaging time, computer storage space, and scoring time.

### 3.2. Effects of 5-Fluorouracil

**Figure 6:**
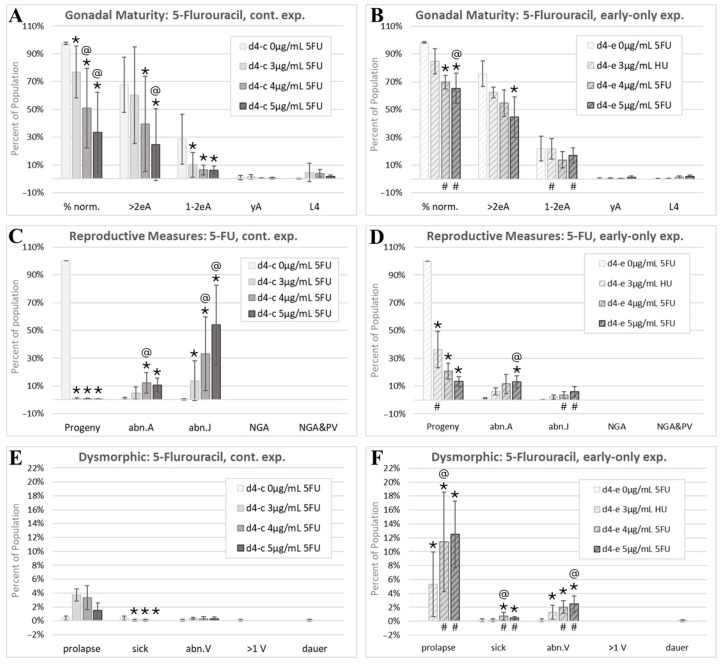
The 5-fluorouracil morphology analysis. Cohorts continuously exposed from the first feeding after hatching through to the first day of adulthood 4 days later are indicated by d4-c, while early-only (24 h) exposure cohorts are labeled as d4-e. (**A**,**B**) The percentage of normal phenotypes and developmental stages. >2eA are adults with more than two internal eggs, 1–2eA are adults with one or two internal eggs, yA are young adults with identifiable gonad arms, a uterus, and/or oocytes, but no internal fertilized eggs, and L4 individuals have fourth-larval-stage size and features. (**C**,**D**) Progeny ratios and the percentage of phenotypes with reproductive tract dysmorphology. (**E**,**F**) Other dysmorphic phenotypes. Error bars show the standard deviation, and significant changes, determined as described in the Statistical Analysis, Section 2.2, are indicated by the following symbols: an * indicates a significant change from plate-matched control cohorts, an @ indicates a significant change from a lower exposure level, and # indicates a significant difference between continuous and early-only exposure groups.

Continuous 5FU exposures resulted in significant decreases in the percentage of individuals scored as normal (% norm., asterisk indicates a significant change from control, Figure 6A). The reduction in the % norm with increasing 5FU exposures was dose-responsive (@ symbol indicates a significant change from a lower dose, Figure 6A). There was far less experiment-to-experiment variability in the percent of normal individuals in the early-only 5FU exposure cohorts, and the % norm. increased relative to continuous exposures (# symbols) but was still significantly lower than in matched control cohorts (Figure 6B). The percentage of normal adults with more than two internal eggs (>2eA) and adults with one or two internal eggs (1–2eA) in each cohort decreased with continuous 5FU exposures (Figure 6A). The percentages of phenotypically normal young adults (yAs) and fourth-larval-stage *C. elegans* (L4s) were small and did not change significantly with 5FU for either exposure scheme (Figure 6B).

The ratio of the number of progeny to the total of those in the parental cohort (Progeny) dropped to nearly zero at all three assessed continuous 5FU exposures (Figure 6C). Note that this reduction was in viable hatched progeny, as 5FU did not appear to interfere with the egg production or laying at the assessed timepoint (Figure 2G,H). With early-only 5FU exposures, there was some, but not complete, recovery of progeny ratios, with the lowest exposure group recovering more than higher exposure groups (Figure 6D). The percent of young adult- and adult-sized individuals with abnormal internal gonadal structures (abn.A) increased to about 10% with both continuous and early-only 5FU exposures and was not statistically different between the two exposure schemes (Figure 6C,D). In contrast, juvenile-sized individuals with gonadal abnormalities (abn.J), mostly abnormal L4s, increased dramatically with continuous 5FU exposures, though this measure was highly variable from one experiment to the next (large abn.J standard deviations, Figure 6C). The incidence of abnormal juveniles was significantly reduced with early-only 5FU exposures, which is consistent with the partial recovery from the growth inhibition and gonadal defects with the removal of 5FU (# symbols, Figure 6D). No individuals lacking gonad arms were identified in the scored 5FU images.

There was a non-significant increase in the incidence of prolapsed individuals continuously exposed to 5FU (Figure 6E). In early-only 5FU exposure groups, the prolapse phenotype was significantly increased above matched controls and continuously exposed cohorts (Figure 6F). This increase may be due to egg-laying contributing to the manifestation of the prolapse phenotype and a higher percentage of individuals reaching the egg-laying stage in the early-only exposure groups. Similarly, the small but significant decreases in the incidences of sick individuals with continuous 5FU exposures (Figure 6E) may be related to the manifestation of this phenotype in adults, not juveniles. The incidence of individuals with an abnormal vulva (abn.V) increased in early-only, but not in continuous, 5FU exposure groups (Figure 6E,F). This again suggests that the increased maturity with early-only exposures can allow for the visualization of abnormal phenotypes that manifest in later stages of development. The single control multi-vulval individual identified in this study (Figure 4D) was one of the 5FU matched controls (Figure 6E). 5FU did not increase the incidence of dauers.

### 3.3. Effects of Hydroxyurea

**Figure 7:**
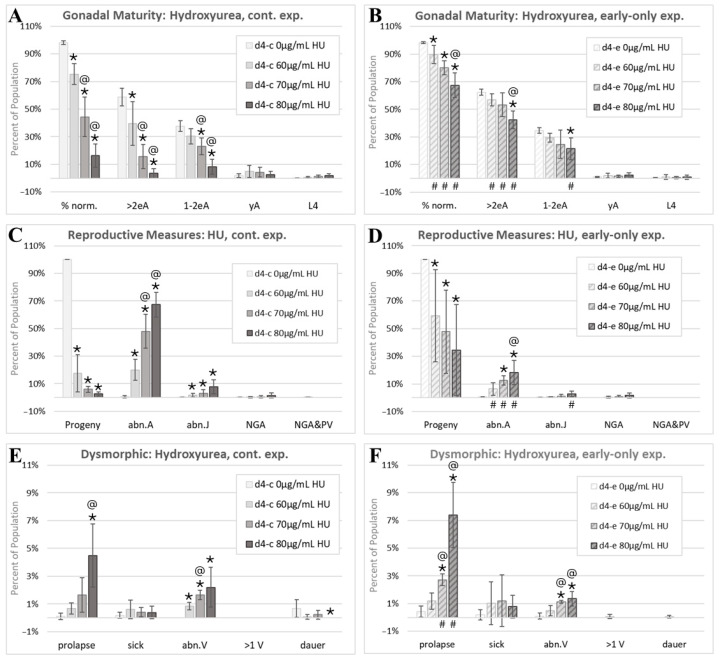
The hydroxyurea morphology assessment. Cohorts continuously exposed from first feeding after hatching through to the first day of adulthood 4 days later are indicated by d4-c, while early-only (24 h) exposure cohorts are labeled as d4-e. (**A**,**B**) The percentage of normal phenotypes and developmental stages. (**C**,**D**) Progeny ratios and the percentage of phenotypes with reproductive tract dysmorphology. (**E**,**F**). Other dysmorphic phenotypes. Error bars show the standard deviation, and significant changes, determined as described in Statistical Analysis, Section 2.2, are indicated by the following symbols: an * indicates a significant change from plate-matched control cohorts, an @ indicates a significant change from a lower exposure level, and # indicates a significant difference between continuous and early-only exposure groups.

The continuous HU exposure induced a sharp dose–response reduction in the percentage of *C. elegans* with a normal morphology of any stage (Figure 7A). Early-only HU exposures significantly increased portions of the populations that remained in the normal category relative to continuous HU exposures (# symbols, Figure 7B), indicating partial reversibility of dysmorphic effects with the removal of HU after 24h of post-hatching development. The progeny output of continuously HU-exposed cohorts was greatly reduced (Figure 7C). There was a trend towards improved progeny production with early-only HU exposures; however, the change did not reach statistical significance due to the inter-experiment variability in progeny-to-adult ratios (Figure 7D) that was not seen with 5FU or RV. There was a dose–response increase in the incidence of adults with an abnormal gonadal morphology (abn.A) with the continuous HU exposure that was significantly reduced, but still present, after removal of HU at 24 h (Figure 7C,D). The increase in the number of abnormal juveniles (abn.Js) was also significant with continuous HU exposures (Figure 7C) but at a far lower incidence than seen with 5FU (Figure 6C). This suggests that continuous HU exposures block normal gonadal development at a later stage than 5FU.

HU induced an increase in prolapse in both the continuous and early-only HU exposure groups (Figure 7E,F), which is consistent with an irreversible developmental effect. There was also an increase in the percentage of individuals with an abnormal vulva (abn.V) with both HU exposure schemes (Figure 7E,F). Of a total of 9880 HU-exposed individuals scored, 1 was scored as multi-vulval (>1 V). This is consistent with the single >1 V individual found among all control groups and an incidence of spontaneous mutations or alterations affecting the vulval number in about one in ten thousand *C. elegans*.

### 3.4. Effects of Ribavirin

**Figure 8:**
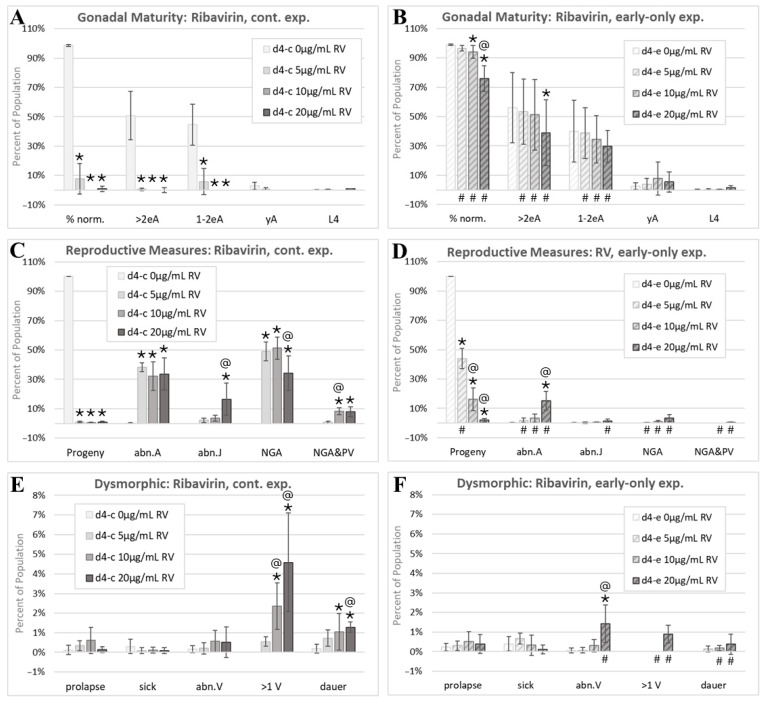
The ribavirin morphology assessment. Cohorts continuously exposed from first feeding after hatching through to the first day of adulthood 4 days later are indicated by d4-c, while early-only (24 h) exposure cohorts are labeled as d4-e. (**A**,**B**) The percentage of normal phenotypes and developmental stages. (**C**,**D**) Progeny ratios and the percentage of phenotypes with reproductive tract dysmorphology. (**E**,**F**) Other dysmorphic phenotypes. Error bars show the standard deviation, and significant changes, determined as described in Statistical Analysis, Section 2.2, are indicated by the following symbols: an * indicates a significant change from plate-matched control cohorts, an @ indicates a significant change from a lower exposure level, and # indicates a significant difference between continuous and early-only exposure groups.

Continuous RV exposures resulted in the near-complete loss of a normal morphology (Figure 8A). There was a significant and substantial recovery of a normal morphology in the early-only RV exposure cohorts, though the recovery was not complete, especially at the highest 20 µg/mL RV early-only exposure level (Figure 8B). Neither eggs nor viable progeny were produced at 5 to 20 µg/mL continuous RV exposures (Figure 8C). At 5 µg/mL RV, but not the two higher exposures, there was a significant, but not complete, recovery of the progeny output with the RV removal at 24 h of post-hatching development (Figure 8D). Morphology and progeny ratios were recorded at a single timepoint in this study. Given the recovery of a mature reproductive tract morphology with early-only RV exposures, a further recovery of the reproductive output with time would be consistent with findings. The majority of continuously RV-exposed individuals were either adults with dysmorphic reproductive structures (abn.A) or no visible gonad arms (NGA), while less than 10% on average were NGA with a protruding vulva (NGA&PV, Figure 8C). The incidences of abn.A, abn.J, NGA, and NGA&PV phenotypes were all significantly reduced in early-only RV exposure groups (# symbols, Figure 8D).

In contrast to 5FU and HU, RV exposures were not associated with significant increases in prolapse (Figure 8E,F). The incidence of abn.V individuals increased at the highest tested early-only RV exposure (Figure 8F). Continuous RV exposures induced a significant dose-responsive increase in multi-vulval (>1 V) individuals (Figure 8E). With early-only exposures, this adverse effect on vulval development was reversed at 5 µg/mL and 10 µg/mL RV and reduced at 20 µg/mL RV (Figure 8F). Dauers made up about 1% of all individuals continuously exposed to 20 µg/mL RV (Figure 8E).

## 4. Discussion

5-fluorouracil (5FU), hydroxyurea (HU), and ribavirin (RV) are antiproliferative drugs that inhibit nucleotide synthesis and repair but by different mechanisms. 5FU inhibits thymidylate synthetase, and can be incorporated into DNA and RNA, resulting in the inhibition of DNA synthesis, DNA damage, and mistranslated proteins [33,34]. HU blocks DNA synthesis and repair by inhibiting ribonucleotide reductase, the enzyme that converts ribonucleotides into deoxyribonucleotides [35]. RV inhibits the synthesis of guanine nucleotides, essential building blocks for DNA and RNA, and blocks mRNA capping, leading to impaired translation [36]. Accurate DNA transcription and repair are critical components of morphogenesis and organogenesis [37,38]. The machinery for DNA replication, damage response, and repair, as well as messenger RNA transcription and modification, are all highly conserved between *C. elegans* and humans [39,40,41], which is consistent with an organism that can model adverse effects from chemicals that interfere with these functions.

Apoptosis is another well-studied and highly conserved pathway that is critical for mammalian and *C. elegans* morphogenesis [42,43]. Other mechanisms of mammalian teratogenesis include endocrine disruption, folate antagonism and interference with neural tube formation, HOX gene expression pattern interference, neural crest cell disruption, oxidative stress, and vascular disruption, as well as interference with molecular modification pathways, such as the renin–angiotensin system, histone deacetylation, and N-methyl-D-aspartate receptor (NMDAR) signaling [44]. As summarized in Table 2, all of these pathways have at least some analogous components in *C. elegans*. Thus, while a simple organism like *C. elegans* cannot model neural tube, heart, or skeletal defects, conserved elements of key pathways indicate that chemicals that adversely alter analogous functions will have apical adverse effects that can be used for hazard identification. Species differences in their pathway genetics, body plan, and developmental timing indicate that chemicals that alter evolutionarily conserved processes critical to animal morphology in non-mammalian systems will not always produce a one-to-one phenocopy of an adverse mammalian outcome; however, this can also be the case with mammalian interspecies comparisons and strengthens the case for better predicting human adverse outcomes by integrating data from a variety of developmental endpoints in multiple models [2,4].

5FU is a chemotherapeutic agent that targets thymidylate synthase and induces reproductive toxicity via germline apoptosis in rodents and in *C. elegans* [107,108,109]. In this study, continuous 5FU exposure from the first feeding after hatching to adulthood reduced the *C. elegans* progeny output by 98% or more (Figure 6C), which is consistent with the abnormal appearance of most laid eggs in these exposure groups (Figure 2G,H). In female mice, ovarian function and oocyte quality recover after the withdrawal of the 5FU administration [110,111,112], and we found that *C. elegans’* reproductive output recovered in early-only 5FU exposure cohorts, though the recovery was reduced with higher early exposures (Figure 6D). While the recovery of the reproductive output was not complete, the progeny production was evaluated at a single timepoint, and the reduced incidence of juveniles with abnormal gonadal structures (abn.J) in the early-only relative to the continuous 5FU exposure groups (Figure 6C,D) suggests that an evaluation at a later timepoint would have shown further reproductive recovery.

In mammals, 5FU exposures are associated with reductions in growth and increases in external fetal malformations, malformations of the reproductive tract, and decreases in oocyte maturity and the weights of both male and female reproductive organs [113,114]. On day four post-initiation of the feeding after hatching, when control *C. elegans* were first-day adults with internal eggs, the most prevalent dysmorphic phenotype observed with continuous 5FU exposures was juveniles with abnormal gonadal development (abn.J, Figure 6C), indicating both reduced whole-body growth and malformations of the reproductive tract, and therefore demonstrates concordance with mammals for these 5FU-induced endpoints. The prevalence of the abn.J phenotype was significantly reduced in 5FU early-only exposure groups (Figure 6D), which is consistent with some adverse 5FU reproductive tract effects being reversible in mammals [113].

In mammals, single maternal injections of 5FU early in development produce later fetal malformations of the digits in mice and eyes in rats [115,116], indicating that the effects on the external morphology of in utero 5FU exposures are irreversible in rodents. In *C. elegans*, 5FU induced intestinal and/or uterine prolapse (Figure 3G,H) in 1.5% to 4% of continuously exposed populations, and the incidence of prolapse increased from 5% to 12% of individuals in early-only 5FU exposure cohorts (Figure 6F), which is consistent with an irreversible effect. The increase in this phenotype in early-only 5FU exposure groups is likely due to the prolapse occurring during egg-laying and the increased proportion of mature *C. elegans* with an intact internal gonadal morphology in the early-only exposure populations (Figure 6). 5FU damages the gastrointestinal tract in rats and mice [117,118]. 5FU is also associated with declines in the muscle mass, grip strength, and muscle fiber size in rats [119]. Therefore, the 5FU-induced prolapse phenotype seen in *C. elegans* could be due to adverse effects on the muscle tissue, including the vulval muscle, damage to the intestine, or both.

Prenatal exposures to HU induce a variety of dysmorphic features in mammals [120], and in *C. elegans* we found a dose–response reduction in the percentage of phenotypically normal individuals of any developmental stage in continuously HU-exposed cohorts (% norm., Figure 7A). Relative to continuous HU exposures, there were significantly higher numbers of normal adults in the early-only HU exposure groups, though there was still a significant dose–response decrease in the percentage of normal phenotypes (Figure 7B), indicating only a partial reversibility of overall HU-induced dysmorphic effects at the concentrations tested.

In mammals, HU induces male reproductive tract anomalies including testicular atrophy and arrested spermatogenesis, but evidence does not support a conclusion of female HU-induced reproductive anomalies [120,121,122]. The most prevalent *C. elegans* abnormality with continuous HU exposures was adults with gonad arm and/or uterine abnormalities (abn.A, Figure 7C). The *C. elegans* reproductive tract develops after hatching from four primordial gonad cells [11]. This timing difference from mammalian fetal reproductive development may explain the species differences in organ-specific effects and underscores the conclusion that apical endpoint effects in simple models have greater potential to predict general categories of toxicity rather than specific mammalian adverse outcomes.

Continuous HU exposures resulted in greatly reduced numbers of progeny relative to the number of individuals in the initially exposed population (Figure 7C). With early-only HU exposures, there was a non-significant trend towards the recovery of progeny production (Figure 7D). A limitation of this study was that effects were only evaluated at a single timepoint; given the increase in adults with a phenotypically normal reproductive morphology in the early-only HU exposure groups (Figure 7B), an assessment of progeny numbers at a second, later timepoint may have detected a greater and more consistent recovery of the reproductive output.

HU induced increases in prolapsed individuals in both continuous and early-only exposure groups (Figure 7E,F), consistent with an irreversible effect. There are indications that HU adversely affects neuromuscular control and induces epithelial degeneration of the gastrointestinal tract in rats [122,123,124], and either of these could be related to the HU-induced prolapse in *C. elegans*.

The antiviral agent RV is a teratogen and reproductive toxicant in rodents, with adverse effects on the morphology and histology of both male and female reproductive organs [125,126,127,128]. RV reversibly inhibits DNA synthesis in mouse embryos, indicating that exposure timing and duration determine RV’s teratogenic effects [128]. In ferrets, the effects of inhaled RV on perinatal growth are also partly reversible [129]. Some adverse effects of RV on the Wistar rat male reproductive tract are reversible while others are not [130].

In *C. elegans* continuously exposed to RV from the first feeding after hatching, the percentage of individuals with a normal reproductive tract morphology was reduced to nearly zero (Figure 8A). The most prevalent dysmorphic phenotype observed with continuous RV exposures was a striking loss of visible gonad arm or uterine structures (NGA) or NGA with a protruding vulva (NGA&PV) (Figure 4A–C and Figure 8C). These two phenotypes were significantly reduced in the early-only RV cohorts (Figure 8D), consistent with reversible RV effects on mammalian reproductive systems.

Continuous RV exposures also induced dose-responsive increases in the incidence of the multi-vulval (>1 V) phenotype (Figure 8E). In early-only exposure groups, multi-vulval individuals were only identified at the highest tested RV concentration and at a reduced incidence relative to continuous exposures (Figure 8D). Continuous RV exposures blocked reproduction (Figure 8C), as would be expected given the observed reproductive tract anomalies. The progeny production recovered slightly with early-only exposures but not at the highest tested concentrations (Figure 8D). 

In summary, in both mammals and *C. elegans*, 5-fluorouracil interferes with thymidylate synthase function and induces germline apoptosis [107,109], inhibits growth [19,115,131], is associated with developmental malformations of reproductive systems (Figure 6C, [113]), and induces a partially reversible reproductive toxicity (Figure 6D, [110,111,112]). The 5FU-induced damage to muscles and intestines reported in rodents [117,118,119] may be related to the irreversible intestinal prolapse effect observed in 5FU-exposed *C. elegans* (Figure 6E,F). In both mammals and *C. elegans*, early hydroxyurea exposure disrupts organismal morphogenesis (Figure 7, [120]). The observed irreversible HU-induced increases in the incidence of intestinal prolapse in *C. elegans* may be related to HU-induced adverse effects in rats on their neuromuscular function and the digestive tract [122,123,124]. In both mammals and *C. elegans*, developmental ribavirin exposures induce growth retardation [19,125] and reproductive malformations (Figure 8, [125]), and some effects on reproduction are at least partially reversible (Figure 8D, [130]).

The examples of genetic homology, analogous functions, and concordant effects discussed here suggest that malformations in *C. elegans* can indicate potential adverse effects in mammals; though given the differences in body plans and the timing of developmental events, these effects are unlikely to be an exact phenocopy. Instead, any chemically induced perturbation in the *C. elegans* growth, morphology, and/or function may indicate an adverse perturbation in mammals, which is consistent with a model that can identify hazardous chemicals to a useful degree. Data from studies using larger and more diverse panels of test chemicals are needed to determine the accuracy and fit-for-purpose use of this model in predicting human hazard.

## Figures and Tables

**Table 1:** Phenotype incidence and statistically significant differences from controls. Incidences for exposed groups are the total of each phenotype over four independent experiments divided by the total scored worms in each group (# assessed). The control incidence is for all continuous and early-only control groups combined. The statistical significance was determined as described in Section 2.2. Symbols indicate that values are statistically (*) different from control, (@) different from a lower exposure, and/or (#) the early-only exposure is different from continuous exposure at the same concentration. Concentrations tested were determined previously as the three highest that were non-lethal and did not fully disrupt the synchronous developmental timing within continuously exposed cohorts [19].

	All Controls	Continuous Exposures									Early-Only Exposures								
		5FU			HU			RV			5FU			HU			RV		
µg/mL	0	3	4	5	60	70	80	5	10	20	3	4	5	60	70	80	5	10	20
# assessed	9150	1379	1314	1334	1557	1500	1566	1640	1247	1305	1357	1819	1692	1749	1604	1620	1779	1247	1305
% norm.	0.9835	0.782 *	0.524 * @	0.343 * @	0.746 *	0.430 * @	0.152 * @	0.069 *	0 *	0.010 *	0.846	0.695 * #	0.650 * @ #	0.903 * #	0.802 * #	0.672 * @ #	0.966 #	0.942 * #	0.756 * @ #
>2eA	0.6149	0.624	0.414 *	0.254 * @	0.385 *	0.149 * @	0.029 * @	0.005 *	0 *	0 *	0.623	0.544	0.440 *	0.568 #	0.532 #	0.423 * @ #	0.543 #	0.520 #	0.388 * #
1-2eA	0.3499	0.096 *	0.062 *	0.064 *	0.305	0.226 * @	0.079 * @	0.051 *	0 *	0 *	0.215 #	0.136	0.171 #	0.297	0.248	0.214 * #	0.382 #	0.341 #	0.295 #
yA	0.0163	0.014	0.005	0.005	0.049	0.041	0.023	0.009	0	0	0.006	0.002	0.013	0.020	0.015	0.025	0.037	0.078	0.055
L4	0.0021	0.043	0.037	0.017	0.006	0.011	0.018	0.004	0	0.010	0.002	0.011	0.021	0.011	0.005	0.009	0.003	0.003	0.016 * @ #
L3	0.0003	0.005	0.005	0.003	0	0.002	0.003	0	0	0	0	0.001	0.005	0.006	0.002	0.002	0.001	0	0.002
male	0.0009	0.002	0	0	0	0.001	0	0	0.001	0	0.001	0.001	0.001	0	0.001	0.001	0	0.001	0.002
abn.A	0.0061	0.045	0.123 * @	0.107 *	0.207 *	0.491 * @	0.681 * @	0.386 *	0.324 *	0.328 *	0.062	0.113	0.133 * @	0.059 #	0.124 * #	0.185 * @ #	0.017 #	0.032 #	0.154 * @ #
abn.J	0.0015	0.127 *	0.314 * @	0.530 * @	0.019 *	0.031 *	0.082 *	0.021	0.034	0.173 * @	0.022	0.038 #	0.060 #	0.006	0.012	0.027 #	0.003	0.002	0.015 #
NGA	0.0001	0	0	0	0.003	0.005	0.012	0.495 *	0.515 *	0.346 * @	0	0	0	0.004	0.009	0.017	0.001 #	0.009 #	0.035 #
NGA&PV	0.0000	0	0	0	0.001	0	0	0.007	0.078 * @	0.074 *	0	0	0	0	0	0	0	0 #	0.002 #
prolapse	0.0023	0.037	0.034	0.015	0.006	0.017	0.043 * @	0.003	0.005	0.001	0.054 *	0.118 * @ #	0.126 * #	0.012	0.027 * @ #	0.074 * @ #	0.003	0.005	0.004
sick	0.0026	0.001 *	0.001 *	0 *	0.006	0.004	0.004	0.001	0.001	0.001	0.002	0.008 * @ #	0.004 * #	0.009	0.011	0.008	0.007 #	0.004 @	0.001 @
abn.V	0.0009	0.004	0.003	0.003	0.008 *	0.017 * @	0.022 *	0.002	0.005	0.005	0.013 *	0.021 * #	0.024 * @ #	0.005	0.011 * @	0.014 *	0.001	0.003	0.015 * @ #
>1 V	0.0001	0	0	0	0	0	0	0.005	0.024 * @	0.044 * @	0	0	0	0.001	0	0	0	0 #	0.009 #
dauer	0.0014	0.001	0	0	0.001	0.002	0 *	0.007	0.012 * @	0.013 * @	0	0.001	0	0.001	0	0	0.001	0.002 #	0.004 #
short	0.0003	0.001	0	0.001	0.003	0	0.001	0.002	0.001	0.002	0.002	0.005	0	0.001	0.002	0.001	0.001	0	0.001
truncated	0.0001	0.000	0.001	0.001	0.001	0	0.001	0	0.001	0	0	0	0	0.001	0	0.001	0	0	0
lumpy	0.0001	0	0	0	0	0.001	0	0	0	0	0	0	0	0	0	0	0	0	0.001
abn.Head	0.0000	0	0	0	0	0	0	0	0	0.001	0	0	0	0	0	0	0	0.001	0
abn.Tail	0.0000	0.001	0.001	0	0	0.001	0.001	0.001	0	0.001	0	0.002	0.001	0	0.001	0.001	0	0	0
F/M	0.0000	0	0	0	0	0	0	0	0	0	0	0	0	0	0	0	0	0	0.001

**Table 2:** Overview of some important pathways involved in mammalian morphogenesis, with aspects that are, and are not, conserved in *C. elegans*.

Pathway	Function	Conserved/Concordant/Analogous	Not Conserved
Endocrine Signaling	- Endocrine hormones act as chemical messengers to control development, growth, metabolism, and reproduction.	- Molecular features of signaling by IGF-1, gonadotropin-releasing hormone, thyrotropin-releasing hormone, and thyrostimulin are conserved and regulate growth in mammals and *C. elegans* [45,46]; - Mammalian endocrine disruptors 4-cumylphenol and bisphenol A (BPA) reduce *C. elegans* brood size [47]; - Human and *C. elegans* homologs of estrogen receptor alpha (ERα/NHR-14) and androgen receptor (AR/NHR-69) utilize similar molecular docking configurations and bind to 4-cumylphenol and BPA [47]; - Exogenous estrogen, progesterone, and testosterone alter *C. elegans* expression of genes for nutrient storage, oxidative stress, and P450 metabolism [48]; - BPA increases *C. elegans’* sterility and embryonic lethality via impaired chromosome synapsis, disruption of meiotic DNA repair, and alteration of germline histone modification [49,50]; - *C. elegans* adult germ cell number is increased by developmental exposure to estradiol and BPA but decreased by endocrine disruptor tributyltin [51]; - Tributyltin damages DNA, inhibits germ cell proliferation, and activates conserved apoptotic mediators in *C. elegans* [52]; - Endocrine disruptor nonylphenol induces *C. elegans* behavior changes, oxidative damage, and suppressed expression of genes involved in serotonin synthesis [53].	- Nematodes do not have pituitary, thyroid, or adrenal glands; - Some nuclear hormone receptor gene groups with mammalian endocrine function do not have *C. elegans* homologs, including NR1A thyroid hormone receptors, NR3A estrogen receptors, NR3C 3-ketosteroid receptors, and the progesterone receptor [54].
Folate and Neural Tube Formation	- Folate deficiency in humans is associated with neural tube and heart defects [55]; - Folate is an essential nutrient for all eucaryotes, used in DNA synthesis and biomolecule methylation [56].	- The intestinal folate uptake system in *C. elegans* and humans is similar, with the *C. elegans* genome coding for homologs of human folate hydrolase 1 (FOLH1) and multiple human folate transporters and carriers [57,58]; - Expression of the *C. elegans* ortholog of human reduced folate carrier SLC19A1 (RFC1) is higher in *C. elegans* juveniles than adults and responsive to folate levels in the diet [57]; - Developmental dietary folate deficiency in *C. elegans* results in protruding vulva, underdeveloped gonad arms, and DNA fragmentation and aneuploidy in meiotic cells [56]; - Vertebrate neural tube closure and *C. elegans* gastrulation are regulated by conserved genes that control tissue-specific internalization of surface cells, actomyosin-driven constriction of specific regions, and the establishment and maintenance of adhesions between specific cells [10,59].	- *C. elegans* do not form a neural tube.
Histone Deacetylation	- Histone modification regulates transcriptional activity and thereby plays important roles in embryogenesis [60].	- Humans and *C. elegans* use conserved histone deacetylases (HDACs) to reprogram epigenetic information in gametes [61,62]; - *C. elegans* histone deacetylases 1 and 2 (*hda-1 and hda-2*) are essential for embryonic viability, and different mutations in *hda-1* can result in specific defects in gonadogenesis and neuronal migration [63,64,65]; - BPA exerts adverse effects on reproductive function in mammals and *C. elegans* via histone modification [50,66].	- The *C. elegans* genome does not code for a homolog of human HDAC11, a Class IV HDAC [62].
HOX Gene Cluster Expression, Spatial and Temporal	- In vertebrates, *HOX* genes control development of the central nervous system, skeleton, gastrointestinal tract, reproductive organs, and limbs [67].	- Conserved *Hox* gene clusters control embryonic body patterning via controlled spatial and temporal expression in vertebrates and nematodes [68]. - *C. elegans Hox* gene expression controls progenitor cell tissue specification, neuronal cell survival, neuronal migration, and synapse formation consistent with their neural patterning roles in mammals [69]; - NOTE: some aspects of *C. elegans Hox* gene controlled developmental body patterning, e.g., gonadogenesis, occur after hatching [11,69].	- *C. elegans* lack a skeletal system. - Only four of the seven core *Hox* orthology groups have *C. elegans* homologs [68].
Neural Crest Formation	- The neural crest is a transient structure in vertebrate development, comprising migratory, multipotent cells that give rise to a variety of cell and tissue types, and plays a crucial role in brain development [70].	- The vertebrate embryonic neural crest is induced and controlled by signaling pathways, including BMP (Bone Morphogenetic Protein), FGFs (Fibroblast Growth Factors), Notch, and Wnt [71]; - Conserved *C. elegans* BMP pathway components regulate developmental processes, including body size, male tail development, and mesoderm patterning, as well as lipid homeostasis via conserved insulin-like growth factor 1 (IGF-1) signaling [72,73]; - Developmental migration of M, the *C. elegans* mesoblast progenitor cell, and its descendants relies on the function of FGF and FGF receptor homologs [74]; - *C. elegans* Wnt signaling controls embryonic cell polarity for anteroposterior axis formation, neuronal cell development and migration, and gonadal progenitor cell migration and symmetry [75,76,77]; - Homologs of *Hox* genes initially identified from studies of *C. elegans* genes essential for neuronal migration were later found to also be necessary for the migration of neural crest cells in vertebrates [69].	- *C. elegans* has a simple neural ring around the pharynx rather than a complex brain with lobes and a blood–brain barrier; - *C. elegans* does not form a neural crest during development.
NMDAR Signaling	- NMDAR signaling in mammals regulates neuronal migration, maturation, and synaptogenesis [44].	- *C. elegans* NMDAR subunit homologues NMR-1 and NMR-2 regulate memory retention, forward and backward movement, and foraging behavior [58,78]; - NMDAR antagonist N_2_O alters *C. elegans* locomotion in an *nmr-1*-dependent manner [79].	- *C. elegans nmr-1* mutants are viable and exhibit no gross developmental defects [80].
Notch Signaling	- Notch signaling controls many aspects of metazoan morphogenesis by controlling cell identity determination, proliferation, differentiation, and apoptosis [81].	- In metazoans, the heterodimeric Notch receptor interacts with Delta ligands (Jagged in mammals, LAG-2 in *C. elegans*) at the plasma membrane [82]; - Conserved elements of Notch signaling control *C. elegans* embryonic tissue specification and germline stem cell proliferation, as well as motor neuron transdifferentiation and migration [83,84,85,86]; - In the mammalian ovary and the *C. elegans* germline, Notch signaling plays important roles in stem cell maintenance [82,83]; - The exocyst complex, which regulates vesicle fusion to the plasma membrane, biochemically interacts with Par5 and Notch in *C. elegans* and human cells [83].	- In vertebrates, Notch signaling controls the morphogenesis of tissues and structures that are not present in *C. elegans.*
Oxidative Stress	- Reactive oxygen species (ROS) can play a role in teratogenesis via altered signal transduction and damage to DNA and other macromolecules [87].	- Antioxidant enzymes are highly conserved between humans and *C. elegans*, and exogenous ROS reduce *C. elegans* growth [88,89]; - Adult *C. elegans’* ROS exposure induces robust antioxidant gene expression controlled by conserved transcription factors [90]; - The gene expression response to oxidizers is not as robust in *C. elegans* juveniles as it is in adults [91], suggesting possible greater ROS sensitivity during development; - Mercury-induced malformations of *C. elegans* reproductive structures are ameliorated by pre-treatment with vitamin E and exacerbated by pre-treatment with paraquat [17]; - Ketamine and methamphetamine are ROS inducers associated with developmental malformations in both mammals and *C. elegans* [18,87]; - ROS inducer benzo[a]pyrene adversely affects growth and reproductive endpoints in *C. elegans* and mammals [92,93].	
Renin–Angiotensin System	- The mammalian renin–angiotensin system regulates vascular resistance and electrolyte balance and plays important roles in human embryonic growth, differentiation, and organogenesis [94].	- In worms and humans, angiotensin-converting enzyme (ACE) plays multiple roles in embryonic growth, differentiation, and organogenesis, as well as adult longevity [95]; - *C. elegans’* ACE homolog *acn-1* function is required for juvenile developmental stage progression and adult morphogenesis [96]; - Captopril is an ACE inhibitor, and *C. elegans’* developmental exposure to captopril induces larval arrest [97]; - *C. elegans acn*-1 function is required for captopril-induced increases in *C. elegans* healthspan and lifespan [98].	- While major portions of the ACE gene and its functions in organismal development are conserved, angiotensin biosynthesis and signaling are not present in nematodes [95].
Vasculogenesis and Angiogenesis	- In mammalian embryos, endothelial cells (ECs) assemble into blood vessel networks via vasculogenesis followed by angiogenesis [99].	- Signals for mammalian vascular development and angiogenesis include angiopoietins, β-catenin, and matrix metalloproteases including MMP2 and MMP9, TGF-β, TNF-α, Notch, and vascular endothelial growth factor VEGF-A [99,100]; - The roles of β-catenins in mediating cell adhesion via E-cadherin and in activating canonical Wnt signaling are conserved in mammals and *C. elegans* [101]; - *C. elegans* genes *zmp-4*, K03B8.6, W01F3.2, and Y50D7A.13 are homologs of human MMP2 and MMP9 and are predicted to have metalloendopeptidase activity [58]; - TGF-β signaling pathways are highly conserved at the molecular and functional level, playing critical roles in determining body size and germline maintenance in *C. elegans* and embryonic body patterning and cell specification in mammals [102,103]; - The *C. elegans* PVF-1 is a VEGF homolog that can bind to human VEGF receptors and induce angiogenesis in vertebrate in vitro models [104].	- *C. elegans* do not have a vascular system or heart; - While there are *C. elegans* angiopoietin-like genes, no angiopoietin receptors are found in the *C. elegans* genome [105]; - The *C. elegans* genome contains TNF-like molecules but does not code for a TNF-α homolog [106].

## Data Availability

All original image data is available at dataDryad.org, Dataset DOI: 10.5061/dryad.j3tx95xs9.

## Abbreviations

The following abbreviations are used in this manuscript:

>1 V	more than one vulva
>2eA	a *C. elegans* adult with three or more internal fertilized eggs
1–2eA	a *C. elegans* adult with one or two internal fertilized eggs
5FU	5-fluorouracil
abn.A	adult-sized *C. elegans* with visible gonadal malformation
abn.J	juvenile-sized *C. elegans* with visible gonadal malformation
abn.V	abnormal vulva
CeHM	*C. elegans* Habitation Medium
F/M	*C. elegans* with an oocyte and a male tail
HU	hydroxyurea
L1	first larval stage
L2	second larval stage
L3	third larval stage
L4	fourth larval stage
NGA	no gonad arms
NGA&PV	no gonad arms and a protruding vulva
RV	ribavirin
